# Leukemia inhibitory factor produced by fibroblasts within tumor stroma participates in invasion of oral squamous cell carcinoma

**DOI:** 10.1371/journal.pone.0191865

**Published:** 2018-02-14

**Authors:** Yae Ohata, Maiko Tsuchiya, Hideaki Hirai, Satoshi Yamaguchi, Takumi Akashi, Kei Sakamoto, Akira Yamaguchi, Tohru Ikeda, Kou Kayamori

**Affiliations:** 1 Department of Oral Pathology, Graduate School of Medical and Dental Sciences, Tokyo Medical and Dental University, Tokyo, Japan; 2 Department of Oral and Maxillofacial Surgery, Graduate School of Medical and Dental Sciences, Tokyo Medical and Dental University, Tokyo, Japan; 3 Department of Maxillofacial Surgery, Graduate School of Medical and Dental Sciences, Tokyo Medical and Dental University, Tokyo, Japan; 4 Department of Surgical Pathology, Graduate School of Medical and Dental Sciences, Tokyo Medical and Dental University, Tokyo, Japan; 5 Department of Oral Health Science Center, Tokyo Dental College, Tokyo, Japan; King Faisal Specialist Hospital and Research Center, SAUDI ARABIA

## Abstract

The interaction between cancer cells and the cancer stroma plays a crucial role in tumor progression and metastasis in diverse malignancies, including oral cancer. However, the mechanism underlying this interaction remains incompletely elucidated. Here, to investigate the interaction between oral cancer cells and fibroblasts, which are major cellular components of the tumor stroma, we conducted an *in vitro* study by using human oral squamous cell carcinoma (OSCC) cell lines and normal human dermal fibroblasts (NHDFs). The results of transwell assays revealed that the migration and invasion of 2 OSCC cell lines, HO1-N-1 and HSC3, were markedly stimulated upon coculturing with NHDFs. To investigate the factors that promote tumor invasion, we isolated NHDFs from cocultures prepared with HO1-N-1 cells and performed microarray analysis. Among the various genes that were upregulated, we identified the gene encoding leukemia inhibitory factor (LIF), and we focused on LIF in further analyses. We confirmed that all OSCC-derived conditioned media potently upregulated LIF expression in NHDFs, and the results of our transwell analysis demonstrated that NHDF-induced OSCC migration and invasion were inhibited by LIF-neutralizing antibodies. Furthermore, immunohistochemical analysis of patient samples revealed that in 44 out of 112 OSCC cases, LIF was expressed in the tumor stroma, particularly in cancer-associated fibroblasts (CAFs), and, notably, clinicopathological analyses confirmed that LIF expression in CAFs was significantly correlated with increased depth of tumor invasion. Collectively, our results suggest that OSCC stimulates fibroblasts to produce LIF, which, in turn, participates in cancer-cell invasion. Our finding offers a potential therapeutic strategy targeting the cancer stroma for the treatment of OSCC patients.

## Introduction

Oral cancer is the most common type of head and neck cancer, and is the sixth most common cancer in the world. More than 90% of the cancers in the oral cavity are histologically classified as oral squamous cell carcinoma (OSCC) [1], which typically behaves in an aggressive manner, exhibiting local invasion and early lymph-node metastasis. Although advances have recently been made in therapeutic strategies for oral cancer, the survival rates of patients with oral cancer have not increased markedly in decades [2, 3].

In the tumor progression in various types of malignancies, including oral cancer, a crucial role is played by the cancer stroma, which is composed of fibroblasts, immune cells, capillaries, basement membrane, and the extracellular matrix surrounding the cancer cells. Among these components, the activated fibroblasts present in the cancer stroma, the so-called cancer-associated fibroblasts (CAFs), are dominant components, and numerous studies conducted over the years have revealed that CAFs play a prominent functional role in cancer progression and metastasis by acting through diverse signaling pathways [4, 5]. CAFs can originate from resident fibroblasts in the immediate vicinity of the tumor, circulating mesenchymal stem cells derived from the bone marrow, cancer cells that have undergone epithelial-to-mesenchymal transition (EMT), or endothelial cells in the tumor [4, 5].

The CAFs in OSCC have been investigated in several studies, which have reported that CAFs play a pivotal role in OSCC progression [6]. CAFs in OSCC promote tumor proliferation [7], invasion [8–11], and local recurrence and lymph-node metastasis [8, 12], followed by poor prognosis [8, 12–14]. Moreover, angiogenesis and lymphangiogenesis, which also play a crucial role in cancer progression and metastasis [15, 16], were reported to be associated with CAFs in OSCC [17].

For cancer treatment, therapeutic strategies targeting the cancer stroma have been expected to be promising [18]; however, for advancing stroma-targeted therapy, deep insights into the different factors and organs involved are required because the tumor microenvironment comprises a complex network of diverse cells and signaling pathways. Thus, our aim here was to investigate the interaction between OSCC cells and fibroblasts in order to elucidate the biological significance of the fibroblasts present within the cancer stroma and evaluate the possibility that these cells could serve as a therapeutic target in OSCC. Our results demonstrated that OSCC cells stimulate fibroblasts to produce leukemia inhibitory factor (LIF), which, in turn, participates in cancer-cell invasion; this finding therefore suggests one potential therapeutic target in OSCC.

## Materials and methods

### Cell culture

Four human SCC cell lines derived from the oral cavity, HO1-N-1, Ca9-22, HSC3, and HSC4, were used. A549, a cell line derived from human lung adenocarcinoma, was used as a positive control for LIF [19]. All cancer cell lines were cultured as described before [20]. Briefly, cancer cell lines were maintained in α-minimum essential medium (α-MEM) containing 10% fetal bovine serum (FBS) (Sigma-Aldrich, St. Louis, MO, USA), 50 U/mL penicillin G, and 50 mg/mL streptomycin. HO1-N-1, Ca9-22, and HSC3 were purchased from Japanese Collection of Research Bioresources (Osaka, Japan). HSC4 was established by Dr. Momose at the Department of Oral and Maxillofacial Surgery, Tokyo Medical and Dental University, as described previously [21], and was kindly provided by Dr. Masao Saitoh. Primary normal human dermal fibroblasts (NHDFs) from an adult donor were purchased from PromoCell (C-12302, Heidelberg, Germany), and incubated in Fibroblast Growth Medium 2 (C-23120, PromoCell). A549 cells were purchased from RIKEN Bio Resource Center (Tsukuba, Japan).

### Collection of conditioned medium (CM)

To collect the CM of OSCC cell lines, HO1-N-1, Ca9-22, HSC3, and HSC4 cells were cultured to confluence in 100-mm dishes in α-MEM containing 10% fetal bovine serum (FBS) (Sigma–Aldrich, St. Louis, MO). After washing 3 times with PBS, the cells were cultured for an additional 24 h in 4 mL of the culture medium without FBS supplementation. Subsequently, the culture supernatants were collected and centrifuged at 1500 rpm for 5 min, and then filtered using a 0.22-μm filter unit. The media obtained were used as the CM after 50% dilution in α-MEM containing 10% FBS.

### RNA extraction, RT-PCR, and real-time quantitative-PCR analyses

RNA was extracted from cells by using a NucleoSpin RNA kit (Macherey-Nagel, Düren, Germany) and reverse-transcribed into cDNA according to methods described previously [20]. For RT-PCR amplification of LIF from OSCC cell lines, the targeted sequences were amplified using Tks Gflex DNA polymerase (Takara Bio, Shiga, Japan) and the following protocol: initial denaturation at 94°C for 2 min, followed by 35 cycles of 94°C for 30 s, 62°C for 30 s, and 68°C for 60 s, and a final extension at 68°C for 2 min. PCR products were visualized using agarose-gel electrophoresis. Quantitative real-time PCR was performed using FastStart Essential DNA green master mix (Roche Applied Science, Penzberg, Germany) and a Light Cycler Nano (Roche Diagnostics, Basel, Switzerland). Relative expression was calculated by using the comparative CT method with GAPDH as an internal control. Primer sequences are listed in S1 Table.

### Coculture analyses of cancer cells and NHDFs

NHDFs were incubated for 24 h and then cocultured with cancer cells for 72 h. For real-time PCR analysis, 5×10^4^ NHDFs/well and 5×10^3^ HSC3 cells/well were seeded into 24-well plates. For microarray analysis, 5×10^4^ NHDFs/well and 5×10^3^ HO1-N-1 cells/well were seeded into 6-well plates.

### NHDF isolation from cocultures

NHDFs were isolated from cocultures by using a magnetic-activated cell sorting (MACS) system with anti-Fibroblast Micro Beads (130-050-601, Miltenyi Biotec, Auburn, CA) and an MS column (130-042-201, Miltenyi Biotec) according to the manufacturer’s protocol. HSC3 cells were isolated from cocultures through negative selection.

### Microarray analysis

To compare the relative expression of transcripts between NHDFs cocultured with HO1-N-1 cells and NHDFs cultured alone, microarray analysis was performed using the Human Gene 2.0 ST Array (Affymetrix, Santa Clara, CA). Total RNA was isolated from NHDFs by using a NucleoSpin RNA kit (Macherey-Nagel), and RNA quality was assessed using an Agilent 2100 Bioanalyzer and Agilent RNA 6000 Nanokit (Agilent Technologies, Santa Clara, CA). Total RNA (100 ng/sample) was converted into cDNA and cRNA was synthesized from the cDNA template by performing *in vitro* transcription, and then single-stranded cDNA was regenerated through reverse transcription. Next, fragmented, endo-labeled cDNA was hybridized to the Human Gene 2.0 ST Array, and stained and washed in a GeneChip FluidicsStation 450 (Affymetrix) and scanned using a GeneChip scanner 3000 7G (Affymetrix).

### Migration and invasion assays

Migration and invasion assays were conducted using 24-well 8.0-μm-pore-size Cell Culture Inserts (Corning, #353097; Corning, NY) and a 24-well BioCoat Matrigel Invasion Chamber (Corning, #354480), respectively. NHDFs (1×10^5^/well) were seeded into the lower well, and on the following day, 2×10^4^ HSC3 cells were seeded into the upper chamber. After culturing for 48 h, the migrated or invaded HSC3 cells on the lower surface of the upper chamber were fixed, stained with crystal violet, and counted in 5 microscopic fields per sample. The average number of cells was calculated based on triplicate experiments.

### Clinical specimens

Our study included primary tongue OSCC specimens from 112 patients; the patients’ clinical characteristics are shown in S2 Table. The included cases were selected from among patients who had been treated at the Dental Hospital of Tokyo Medical and Dental University. All tissues were fixed with 10% neutral buffered formalin and embedded in paraffin according to routine laboratory protocols. Histological sections (4 μm thick) were stained with hematoxylin and eosin (H&E) for microscopic examination. All the clinical samples used in the study were anonymized before access by any of the authors. All experimental procedures were approved by the ethics committee of the Faculty of Dentistry, Tokyo Medical and Dental University (registration number D2014-061-01). Because all tissue specimens were originally obtained for diagnostic purposes, the institutional ethics committee consented to waive the requirement for specific informed consent in accordance with the amended Ethical Guidelines for Clinical Studies provided by Ministry of Health, Labor and Welfare of Japan (July 31, 2008). This research plan was disclosed in poster format in the outpatient clinic of the oral surgery department, and thus the patients were provided the opportunity to decline the research use of their pathological samples. This procedure substituted for written informed consent, and this was approved by the ethics committee. The archived tissue specimens were anonymized and used for research.

### Immunohistochemistry

Immunohistochemical staining was performed on formalin-fixed, paraffin-embedded human OSCC sections. The primary antibodies used were α-smooth muscle actin (α-SMA) mouse monoclonal antibody (1:100, M0851; Dako, Glostrup, Sweden) and LIF rabbit polyclonal antibody (1:100, TA321468; OriGene Technologies, Rockville, MD); antigen retrieval was performed according to manufacturer protocols. EnVision+ Dual Link (Dako) was used as the secondary antibody, and coloration was conducted using diaminobenzidine substrate.

For double-immunofluorescence staining, the aforementioned mouse anti-α-SMA (1:100) and rabbit anti-LIF (1:100) antibodies were used as primary antibodies, and the secondary antibodies used were Alexa Fluor 488 goat anti-rabbit IgG (A11008, Invitrogen, Carlsbad, CA) and Alexa Fluor 594 goat anti-mouse IgG (A11005, Invitrogen). Nuclei were stained with DAPI. Immunofluorescence images were captured using an Axioskop 2 plus microscope (Carl Zeiss, Jena, Germany).

### Evaluation of immunohistochemical analyses

Three pathologists without prior knowledge of the patients’ clinicopathological data evaluated the immunohistochemical results obtained for α-SMA and LIF. Evaluation was performed based on the staining intensity and the proportion of “positive” fibroblastic cells in the tumor stroma, according to a previous report [22]. Briefly, staining intensity was divided into four groups: negative, weak, moderate, and strong. Cases exhibiting moderate or strong intensity in >30% of the fibroblasts in the tumor stroma were regarded as positive for the expression of the examined protein.

### Statistical analyses

Statistical analyses were performed using Ekuseru Toukei 2015 (Social Survey Research Information, Tokyo, Japan). Correlations between α-SMA or LIF expression and clinicopathological parameters were analyzed using Fisher’s exact test with Bonferroni correction. In the evaluation of cell-culture experiments, Student’s *t* test was applied to compare the differences in the mean values between two groups. To analyze the differences in the mean values among multiple groups, we used a one-way ANOVA followed by multiple-comparison with Dunnett’s or Tukey’s method. Overall survival rate was estimated according to the Kaplan-Meier method, and the log-rank test was used to analyze the statistical significance. Overall survival times were calculated from the date of initial surgery to the date of death.

## Results

### Fibroblasts promote migration and invasion of OSCC cells

**Fig 1:**
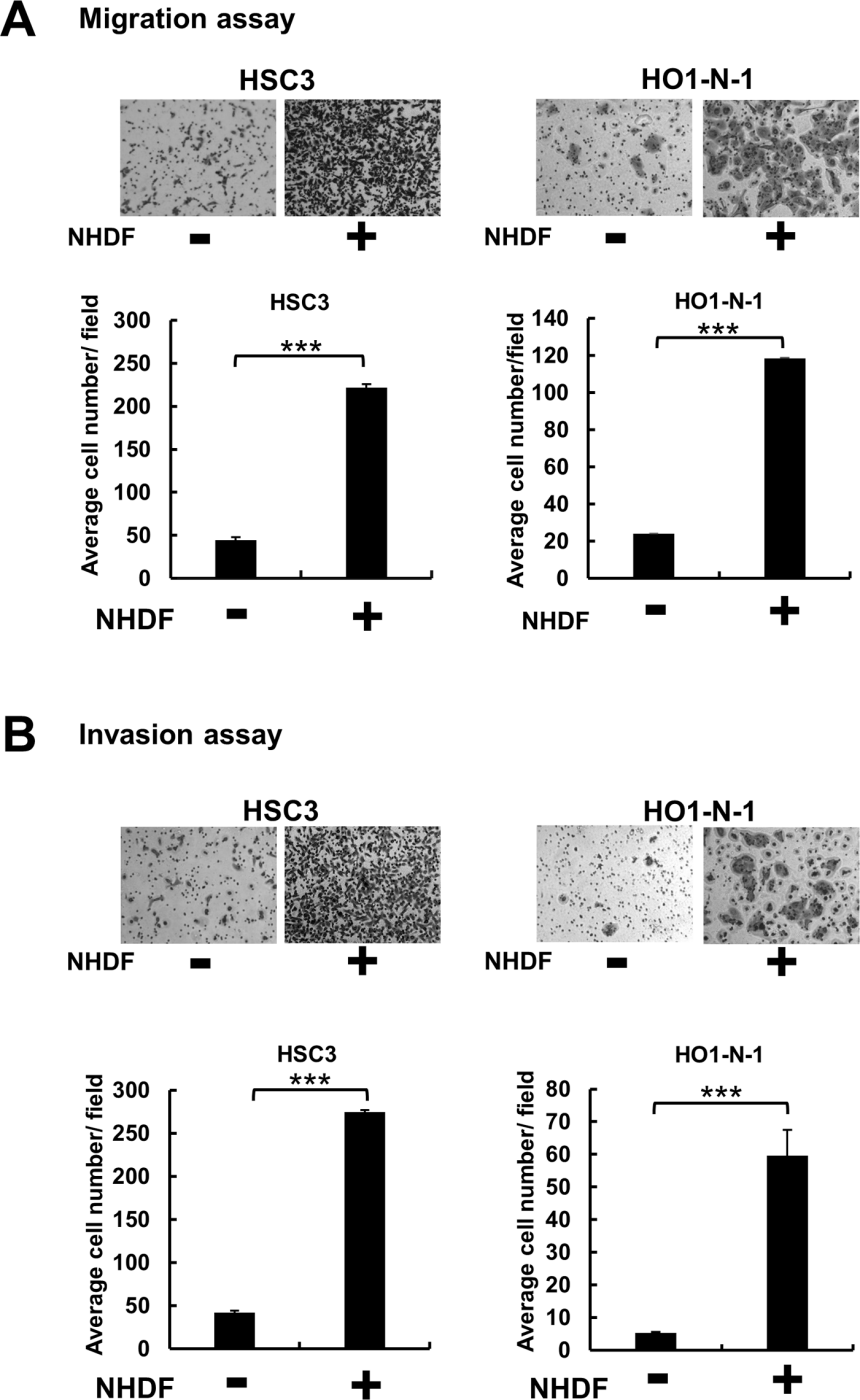
Fibroblasts promote migration and invasion of OSCC cells. Transwell migration (**A**) and invasion (**B**) assays performed using cell-culture inserts. In the upper chamber, 2×10^4^ OSCC cells (HSC3, HO1-N-1) were seeded, and 1×10^5^ NHDFs were seeded into the lower well. After 48 h, the OSCC cells that had migrated or invaded into the lower surface of the upper chamber were stained and quantified. Representative images and graphs of 3 independent experiments are shown. ***p < 0.001 compared with control. Data represent means ± SEM.

To investigate the effect of fibroblasts on cancer progression, we conducted *in vitro* migration and invasion assays. The results of transwell assays revealed that the cells of 2 OSCC cell lines, HSC3 and HO1-N-1, showed significantly higher migration and invasion activities when cultured with NHDFs than when cultured alone (Fig 1A and 1B). These results suggested that fibroblasts exhibit the potential to promote OSCC cell migration and invasion by producing certain soluble factors in response to the cancer cells.

### OSCC induces LIF expression in fibroblasts

**Fig 2:**
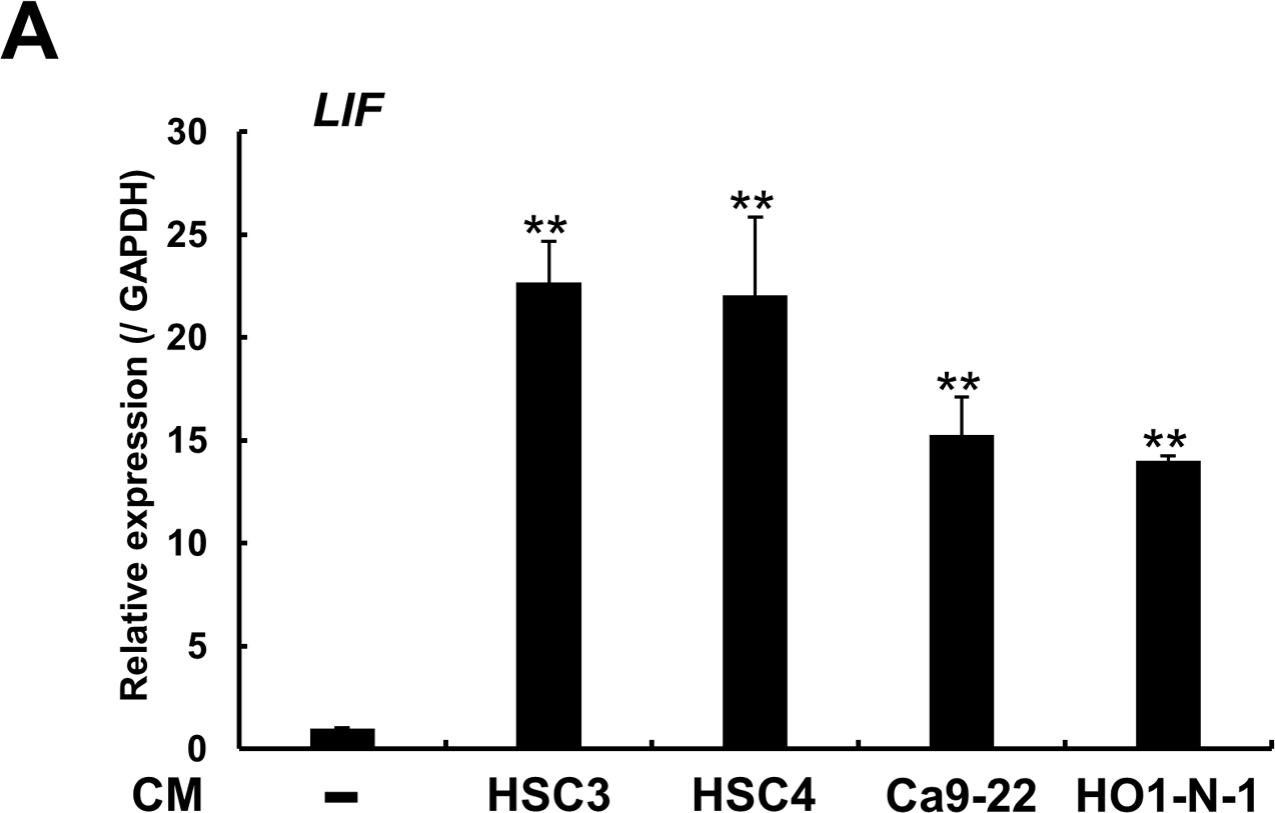
OSCC cell lines stimulate LIF expression in NHDFs. CM derived from 4 OSCC cell lines were separately added to NHDFs, and increased LIF expression was confirmed through quantitative real-time PCR analysis after 48 h of culture. The experiment was performed in triplicate in 24-well plates. Representative graph from 3 independent experiments is shown. Data represent means ± SEM. Multiple comparisons were performed by using one-way ANOVA with Dunnett’s method. **p < 0.01 compared with control NHDFs.

To investigate the factors that promote cancer-cell migration and invasion, we performed microarray analysis and compared the expression profiles of NHDFs cultured alone and NHDFs cocultured with HO1-N-1 cells: In the cocultured NHDFs, 246 genes were upregulated >2-fold relative to their expression in NHDFs cultured alone, and 23 of these genes (Table 1) showed >5-fold upregulation. Maximal upregulation in the cocultured NHDFs was measured for the gene encoding transforming growth factor (TGF)-β2, which has been extensively analyzed by numerous researchers and has been confirmed to induce EMT; however, in this study, we focused on LIF, which exhibited the third-highest upregulation in NHDFs cocultured with HO1-N-1 cells. LIF belongs to the interleukin-6 (Il-6) superfamily, and plays multifunctional roles in neuronal development, embryo implantation, stem cell renewal, inflammation, and immune-system functions. LIF is also recognized to play a critical role in the progression of several types of solid malignancies, including head and neck cancer [23]. To investigate whether other OSCC cell lines induce LIF expression in NHDFs, we obtained the CM from 4 OSCC cell lines and added each CM separately to NHDFs, and then confirmed LIF expression by performing quantitative real-time PCR analysis. All OSCC-derived CM, particularly HSC3 CM, significantly increased LIF expression in NHDFs (Fig 2).

**Table 1:** Genes whose expression was >5-fold higher in cancer-cocultured NHDFs than in normal NHDFs.

Gene symbol	Gene products	Fold-change*
***TGFB2***	Transforming growth factor-β2	18.28
***EDNRA***	Endothelin receptor type A	12.58
***LIF***	Leukemia inhibitory factor	12.31
***POSTN***	Periostin	12.08
***IL24***	Interleukin 24	11.50
***PTGS2***	Prostaglandin-endoperoxide synthase 2	11.13
***CXCL8***	C-X-C motif chemokine ligand 8	8.93
***CPE***	Carboxypeptidase E	7.92
***IER3***	Immediate early response 3	7.45
***SLC16A6***	Solute carrier family 16 member 6	7.09
***OLFM2***	Olfactomedin 2	6.59
***PAG1***	Phosphoprotein membrane anchor	6.47
	with glycosphingolipid microdomains 1	
***PAPPA***	Pappalysin 1	6.38
***IFI44L***	Interferon induced protein 44 Like	6.30
***POM121L9P***	POM121 transmembrane nucleoporin like 9	5.69
***LPPR4***	Lpid phosphate phosphatase-related protein type 4	5.58
***MEDAG***	Mesenteric estrogen dependent adipogenesis	5.56
***NPR3***	Natriuretic peptide receptor 3	5.52
***ABI3BP***	ABI family member 3 binding protein	5.50
***WISP1***	WNT1 inducible signaling pathway protein 1	5.50
***LOC101928188***	Uncharacterized LOC101928188	5.40
***CSF3***	Colony stimulating factor 3	5.39
***DDIT4***	DNA damage inducible transcript 4	5.03

*Cocultured-NHDF/NHDF

### LIF participates in migration and invasion of OSCC cells

**Fig 3:**
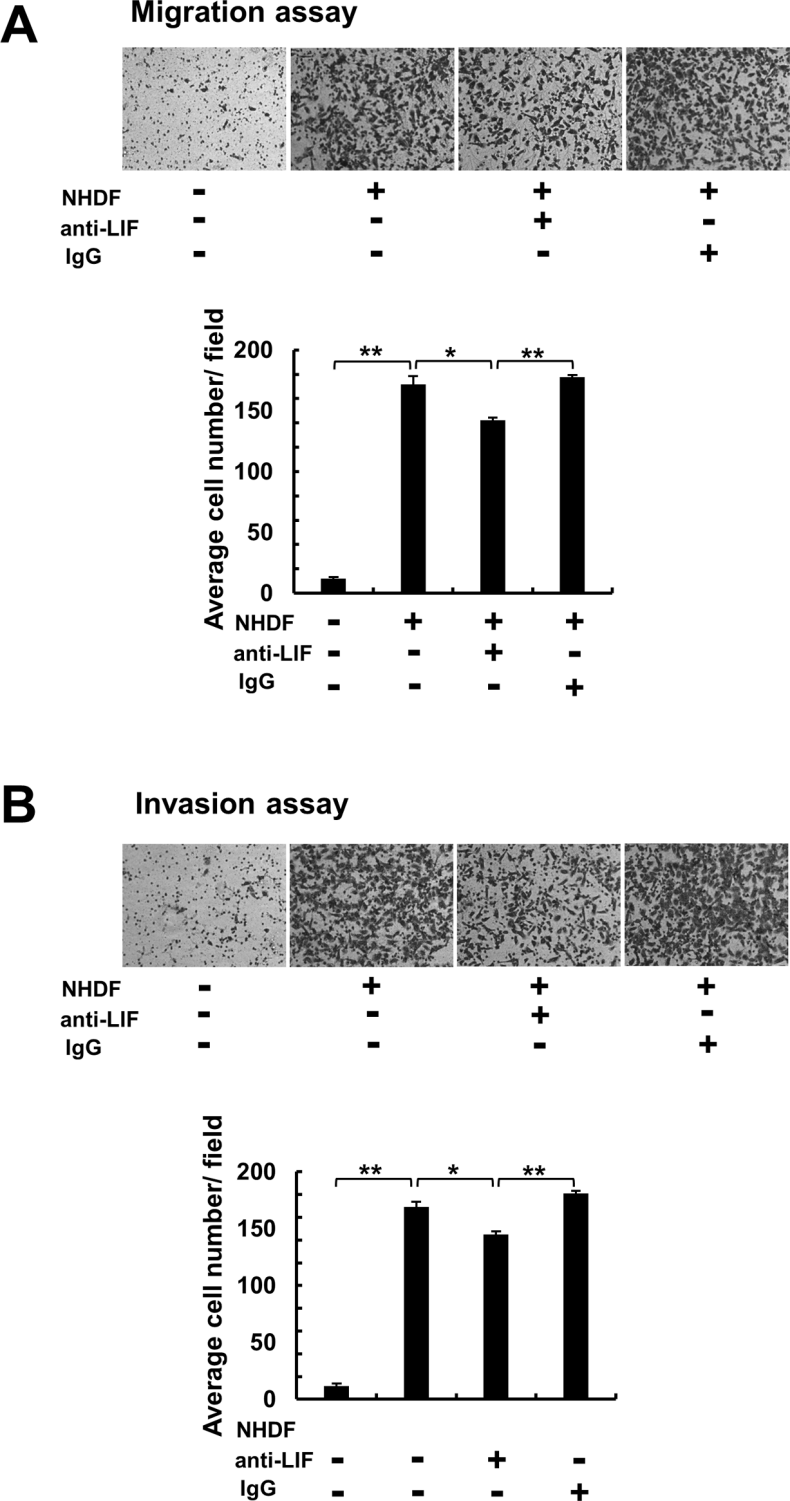
Effect of anti-LIF antibody on migration and invasion of HSC3 cells. Transwell migration (**A**) and invasion (**B**) assays performed using cell-culture inserts. In the upper chamber, 2×10^4^ HSC3 cells were seeded, and 1×10^5^ NHDFs were seeded into the lower well. In each experiment, cells were treated with 0.5 μg/mL of an anti-LIF neutralizing antibody. IgG was used as a control. After 48 h, the migrated or invaded HSC3 cells on the lower surface of the upper chamber were stained and quantified. Representative images and graphs of 3 independent experiments are shown. Multiple comparisons were performed by using one-way ANOVA with Tukey’s method. *p < 0.05, **p < 0.01 compared with control NHDFs. Data represent means ± SEM.

To validate the effect of LIF on OSCC migration and invasion, we conducted *in vitro* studies. First, we used RT-PCR analysis and confirmed that LIF receptor was expressed in OSCC cell lines (S1 Fig). Next, we performed transwell assays, and the results revealed that NHDF-induced migration/invasion activities of the OSCC cell line HSC3 were partially inhibited by treatment with an anti-LIF neutralizing antibody (Fig 3A and 3B). Collectively, our *in vitro* studies demonstrated that OSCC cell lines stimulate LIF production in NHDFs, and that NHDF-derived LIF participates in cancer-cell migration and invasion.

### LIF expression profile in human OSCC tissues

**Fig 4:**
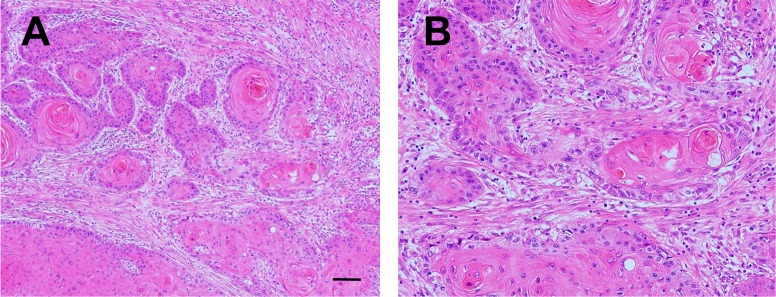
Histology of the tumor invasive region in human OSCC. **A, B:** Representative histological images of human OSCC cases. Desmoplastic reaction was observed around invasive cancer nests. H&E staining. Scale bar: 100 μm (A) and 200 μm (B).

Next, we conducted immunohistochemical analyses to investigate the distribution of LIF expression in the tumor microenvironment by using 112 human tongue OSCC samples. In the tumor invasive front, varying amounts of stromal cells, fibroblasts, inflammatory cells, microvessels, etc., were found to intervene between cancer nests (Fig 4A and 4B).

**Fig 5:**
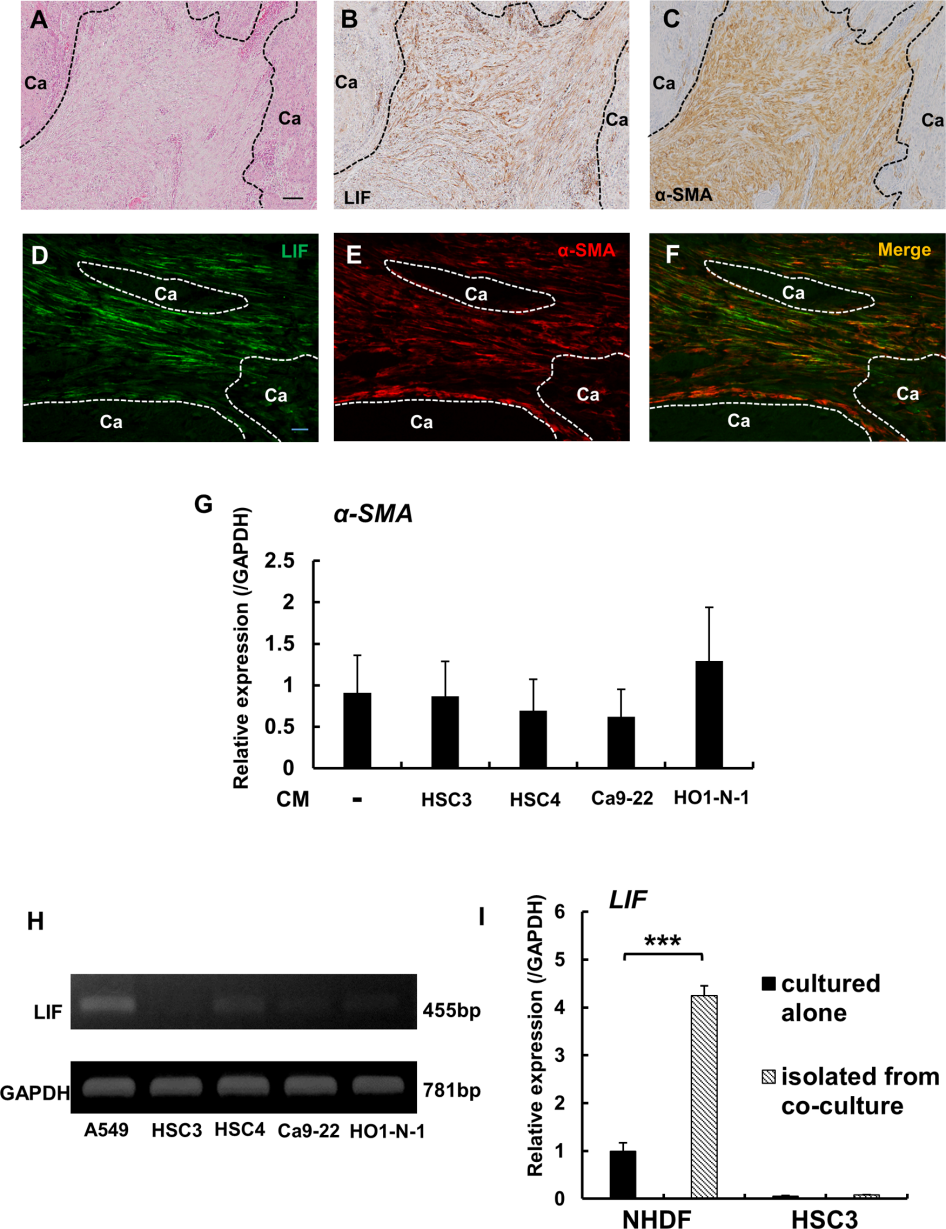
LIF expression in CAFs in OSCC. **A:** Histology of OSCC invasive front (H&E staining). Scale bar: 100 μm. **B:** Distribution of LIF-positive fibroblastic cells within the tumor stroma. **C:** Distribution of α-SMA-positive fibroblastic cells within the tumor stroma. **D-F:** Double-immunofluorescence staining for LIF (green) and α-SMA (red). Most fibroblasts in the cancer stroma showed colocalization of LIF and α-SMA, but some of the LIF-expressing fibroblasts were negative for α-SMA. Scale bar: 100 μm. Dotted lines in A-C and E-G indicate the interface of cancer nests and the stroma. **G:** CM derived from 4 OSCC cell lines were added to NHDFs, and α-SMA expression levels in NHDFs were assessed using quantitative real-time PCR analysis after culturing for 48 h. **H:** LIF expression in human OSCC cell lines. LIF expression was confirmed through RT-PCR analysis. A549, a human lung adenocarcinoma cell line, was used as a positive control. **I:** LIF expression in NHDFs and HSC3 cells in coculture; 5×10^4^ NHDFs and 5×10^3^ HSC3 cells were cocultured in 24-well plates. The experiments were performed in triplicate. After 72 h, NHDFs and HSC3 cells were separately isolated from the cocultures through positive and negative selection by using the MACS system. LIF expression was investigated using quantitative real-time PCR analysis. Data represent means ± SEM. ***p < 0.0001.

In immunohistochemical analysis, LIF was detected in cancer stromal fibroblasts (Fig 5A and 5B). These LIF-positive fibroblasts were colocalized with fibroblasts exhibiting immunoreactivity for α-SMA, a widely recognized marker of CAFs (Fig 5C). Overall, 44 out of 73 α-SMA-positive OSCC cases were LIF-positive, and the remaining 29 cases were LIF-negative (Table 2). To further characterize LIF expression in fibroblasts, we performed double-immunofluorescence staining by using LIF and α-SMA antibodies. Most of the LIF expression was detected in α-SMA-positive CAFs, although LIF was also detected in a few α-SMA-negative fibroblasts (Fig 5D–5F). We also investigated *in vitro* whether OSCC cells stimulate α-SMA expression in fibroblasts, and found that α-SMA expression in NHDFs was not markedly altered by any OSCC-derived CM (Fig 5G).

**Table 2:** Clinicopathological significance of LIF expression in CAFs.

		LIF in CAFs		
Characteristics	Total	Negative, n (%)	Positive, n (%)	p value^c^
**CAF-positive**	73	29 (39.7)	44 (60.3)	
**Age (y)**				
<63^a^	48	29 (60.4)	19 (39.6)	0.0000*
≥63^a^	25	0 (0.0)	25 (100.0)	
**Sex**				
male	16	12 (75.0)	4 (25.0)	0.0029*
female	57	17 (29.8)	40 (70.2)	
**Differentiation**				
Well	32	10 (31.2)	22 (68.8)	0.2326
Mod/Poor	41	19 (46.3)	22 (53.7)	
**Tumor size**				
≤2 cm	6	3 (50.0)	3 (50.0)	0.6762
>2 cm	67	26 (38.8)	41 (61.2)	
**Tumor depth**				
≤5 mm	28	18 (64.3)	10 (35.7)	0.0012*
>5 mm	45	11 (24.4)	34 (75.6)	
**T-stage**^**b**^				
T1	19	12 (63.2)	7 (36.8)	0.0276
T2,3,4,	54	17 (31.5)	37 (68.5)	
**N-stage**^**b**^				
N0	39	15	24	0.826
≥N1	34	14	20	

^a^Mean value

^b^According to the UICC 8th edition TNM classification

^c^By Fisher’s exact test

*Statistically significant based on Bonferroni correction (p < 0.05/7 = 0.0071)

LIF expression in OSCC cells was considerably weaker than that in CAFs, and the results of *in vitro* RT-PCR analyses confirmed the low expression level of LIF in OSCC cell lines (Fig 5H). Next, NHDFs were cocultured with HSC3 cells, and then the NHDFs and HSC3 cells were separately isolated from the cocultures by using the MACS system; subsequently, LIF expression was examined using quantitative real-time PCR analysis (Fig 5I): LIF expression was markedly increased in NHDFs isolated from the cocultures as compared with the expression level in NHDFs cultured alone; by contrast, only very low LIF expression was detected in HSC3 cells either cultured alone or cocultured with NHDFs. Collectively, our data demonstrated that fibroblasts, including CAFs, produced LIF, with CAFs the main sources of LIF in the OSCC microenvironment.

### Clinicopathological significance of LIF in CAFs

To confirm the clinicopathological significance of LIF-positive CAFs, we evaluated the correlation between LIF-positive CAFs and clinicopathological characteristics by using 73 CAF (+) OSCC cases (Table 2) with CAFs defined as α-SMA-positive fibroblasts. LIF-positive CAFs were correlated with older age (p < 0.0001), sex (female) (p = 0.0029), and tumor invasion depth (p = 0.0012).

**Fig 6:**
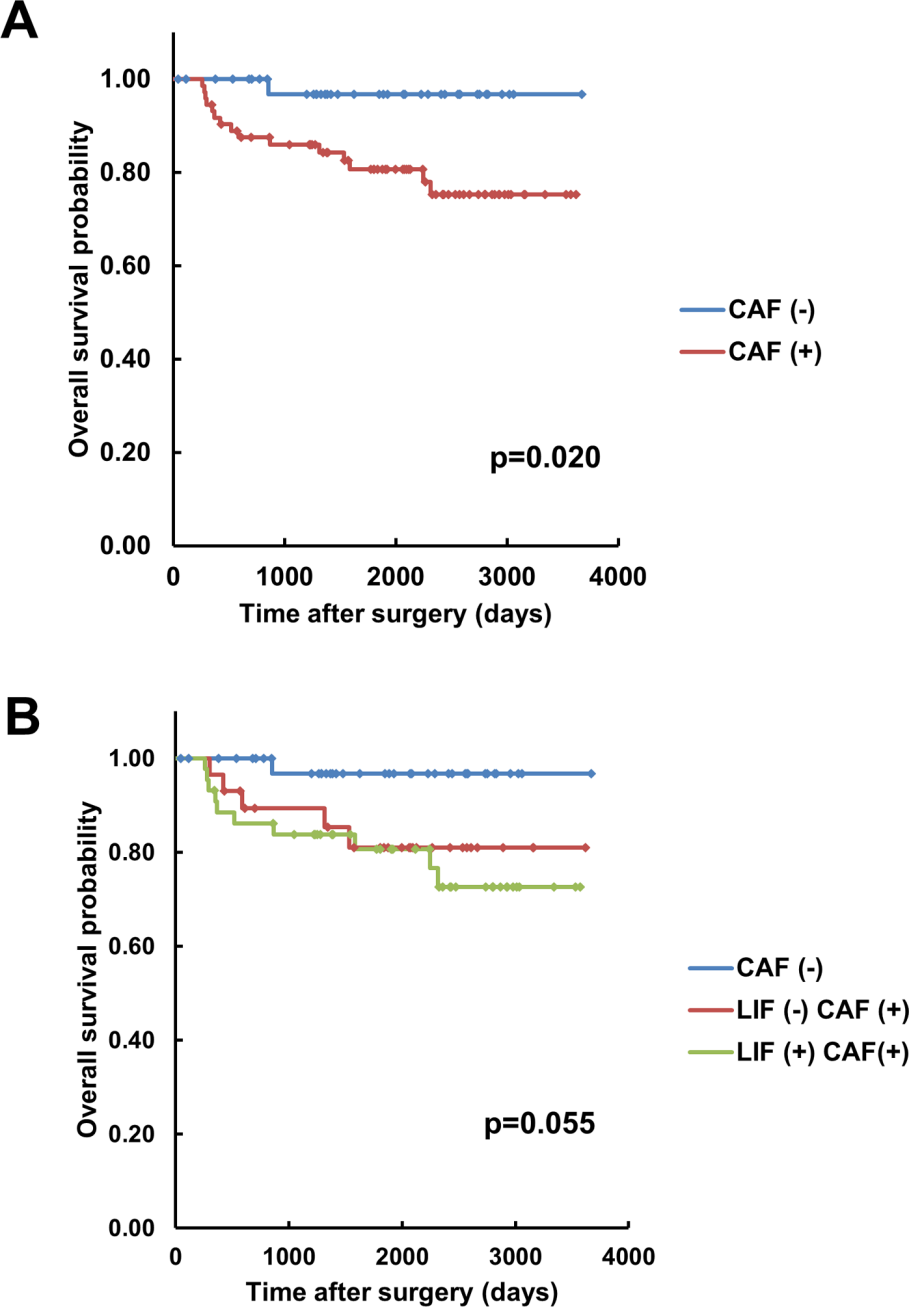
Analysis of overall survival associated with LIF expression in CAFs in human OSCC. **A:** Kaplan-Meier curve for overall survival in relation to the presence of CAFs in 112 human OSCC cases. **B:** Kaplan-Meier curve for overall survival in relation to the presence of LIF in CAFs in 112 human OSCC cases.

Lastly, we evaluated the prognostic value of LIF-positive CAFs. Although the presence of CAFs was significantly correlated with poor overall survival of OSCC patients (p = 0.020) (Fig 6A), the presence of LIF-positive CAFs showed no significant prognostic value in terms of the overall survival of OSCC patients (Fig 6B).

## Discussion

In this study, we analyzed the interaction between OSCC cells and fibroblasts and found that NHDFs stimulated the migration and invasion of OSCC cells. Because the NHDFs used were normal fibroblasts, we speculated that the molecules that stimulated OSCC cell migration and invasion were upregulated in the NHDFs that interacted with OSCC cells. In NHDFs cocultured with OSCC cells, TGF-β and LIF were markedly upregulated. TGF-β has been widely shown to function as a key molecule that induces EMT in cancer cells. On the other hand, LIF is a multifunctional cytokine belonging to IL-6 family that performs critical biological functions in cell proliferation and differentiation.

IL-6 is a well-known inflammatory cytokine that promotes various biological activities through its binding to IL-6 receptors. Additionally, IL-6 is produced by CAFs and promotes tumor-cell migration and invasion [24, 25], angiogenesis [26], and chemoresistance [27] in various types of malignancies. Kayamori et al demonstrated that IL-6 produced by stromal fibroblasts associated with OSCC stimulated osteoclastic bone resorption [20]. LIF promotes biological activity through binding to its specific receptor, LIF receptor-α, followed by formation of a heterodimer with the GP130 signal-transducing subunit and activation of JAK/STAT and MAPK-signaling cascades [28]. LIF is reportedly overexpressed in several kinds of human malignancies, including head and neck cancer [19, 29–33]; however, its expression in the cancer microenvironment is largely unknown. Therefore, in this study, we focused on analyzing the expression of LIF in CAFs.

Our study showed that an anti-LIF neutralizing antibody partially reversed the stimulation of migration and invasion of OSCC cells cocultured with NHDFs. These results not only suggested that LIF participated in the stimulation of OSCC cell migration and invasion, but also that other factors, particularly TGF-β, were involved in this stimulation. Intriguingly, our *in vitro* analysis revealed that OSCC-derived CM stimulated LIF expression, but not α-SMA expression, in NHDFs. We previously showed that the expression of α-SMA was upregulated in NHDFs cocultured with OSCC cells [22]. Collectively, these data suggest that direct contact between OSCC cells and NHDFs was necessary for inducing α-SMA expression in NHDFs; this implies that in the human OSCC tissues, stromal fibroblasts in contact with OSCC cells express α-SMA, and that these fibroblasts express LIF upon stimulation by certain soluble factors expressed by the OSCC cells. Here, our double-immunofluorescence staining of human OSCC samples revealed that the majority of LIF-positive fibroblasts expressed α-SMA, and thus the main source of LIF was considered to be the α-SMA-positive cells. The double-staining results also showed that α-SMA-positive cells were not unfailingly in contact with OSCC cells. Several CAF markers exist, and the expression pattern of CAF markers is reported to vary depending on individual cases [4, 5, 34]. However, α-SMA is the most common marker used to identify CAFs [4, 5], and thus we considered the α-SMA-positive cells to be identical to CAFs.

Given the findings of our previous study [22], we suggest that OSCC cells exhibit the potential to induce the differentiation of stromal fibroblasts into CAFs, although other mechanisms of CAF induction also exist.

The mechanism underlying the preferential expression of LIF in CAFs remains unclear. Albrengues *et al* reported that TGF-β stimulated LIF production in tumor cells and fibroblasts, and further demonstrated that LIF mediated TGF-β-dependent actomyosin contraction and subsequent extracellular matrix remodeling, which led to cancer-cell invasion *in vitro* and *in vivo* [19]. These intriguing data potentially support our results, but the precise role of LIF in the cancer microenvironment and its relation to fibroblasts remain incompletely elucidated, and should be further analyzed in future studies by using CAFs.

Ha *et al* investigated 116 esophageal squamous cell carcinoma (ESCC) specimens for the expression of 5 CAF markers: fibroblast activation protein, fibroblast-specific protein-1 (FSP1), platelet-derived growth factor receptor (PDGFR) α, PDGFRβ, and α-SMA; the results suggested that FSP1, PDGFRα, and α-SMA were unfavorable prognostic indicators of ESCC [34]. Kellermann *et al* reported that in OSCC cases, the abundant presence of myofibroblasts, particularly at the invasive tumor front, was significantly associated with shorter overall survival [12]. In accord with these reports and other studies on OSCC [8, 13, 14], our clinicopathological analysis here revealed that the presence of α-SMA-positive CAFs was significantly correlated with poor prognosis. However, we detected no significant prognostic difference between LIF (+) CAF and LIF (-) CAF cases. Immunohistochemical and double-immunofluorescence staining analyses revealed that α-SMA and LIF exhibited similar expression profiles. Therefore, we do not consider the result of the statistical analysis of OSCC cases to be indicative of any contradiction in terms of α-SMA and LIF expression and the prognosis for OSCC patients. To evaluate the prognostic value of LIF expression in cancer stromal cells, further analysis must be conducted using additional OSCC cases. Intriguingly, the results of immunohistochemical analysis of 50 head and neck cancer samples by Albrengues *et al* indicated that elevated LIF expression was significantly correlated with poor clinical outcomes [19]; however, based on immunohistochemical evaluation, LIF expression was reported to be preferentially detected in cancer tissues rather than in the cancer stroma [19]. Conversely, our immunohistochemical staining for LIF revealed that LIF expression was considerably lower in cancer cells than in CAFs, and that in >50% of the cases, the cancer cells were LIF-negative. The reason for these discrepancies is unclear, but our *in vitro* study revealed that LIF expression in multiple OSCC cell lines was substantially lower than that in NHDFs, particularly in NHDFs stimulated by OSCC cells. The results of both our *in vitro* and *in vivo* studies clearly showed that LIF was predominantly expressed by fibroblastic cells rather than OSCC cells, but further analysis is required to clarify the expression profile of LIF in cancer tissues.

In conclusion, we demonstrated that OSCC cells stimulated fibroblasts to produce LIF, and the fibroblast-derived LIF, in turn, mediated cancer-cell migration and invasion. Moreover, our results indicated that CAFs were the major source of LIF in OSCC. Our findings provide new insights into the interaction between cancer cells and the cells of the cancer stroma and identify a potential target for OSCC therapy.

## Supporting information

S1 Fig OSCC cells express LIF receptor. LIFRα expression in OSCC cells was detected using RT-PCR. The targeted sequences were amplified using Prime STAR GXL DNA Polymerase (Takara Bio, Shiga, Japan) and this amplification protocol: initial denaturation at 94°C for 2 min, followed by 35 cycles of 94°C for 30 s, 55°C for 1 min, and 72°C for 90 s, and a final extension at 72°C for 2 min. Primer sequences are shown in S1 Table.
(TIF)

S1 Table Primers used for PCR amplification. (XLSX)

S2 Table Clinicopathological characteristics of OSCC patients. (XLSX)
