# Supplementary figures and images for: Leukemia inhibitory factor produced by fibroblasts within tumor stroma participates in invasion of oral squamous cell carcinoma
Source: PLoS One. 2018 Feb 14;13(2):e0191865. doi: 10.1371/journal.pone.0191865 (PMC5812599; doi:10.1371/journal.pone.0191865)

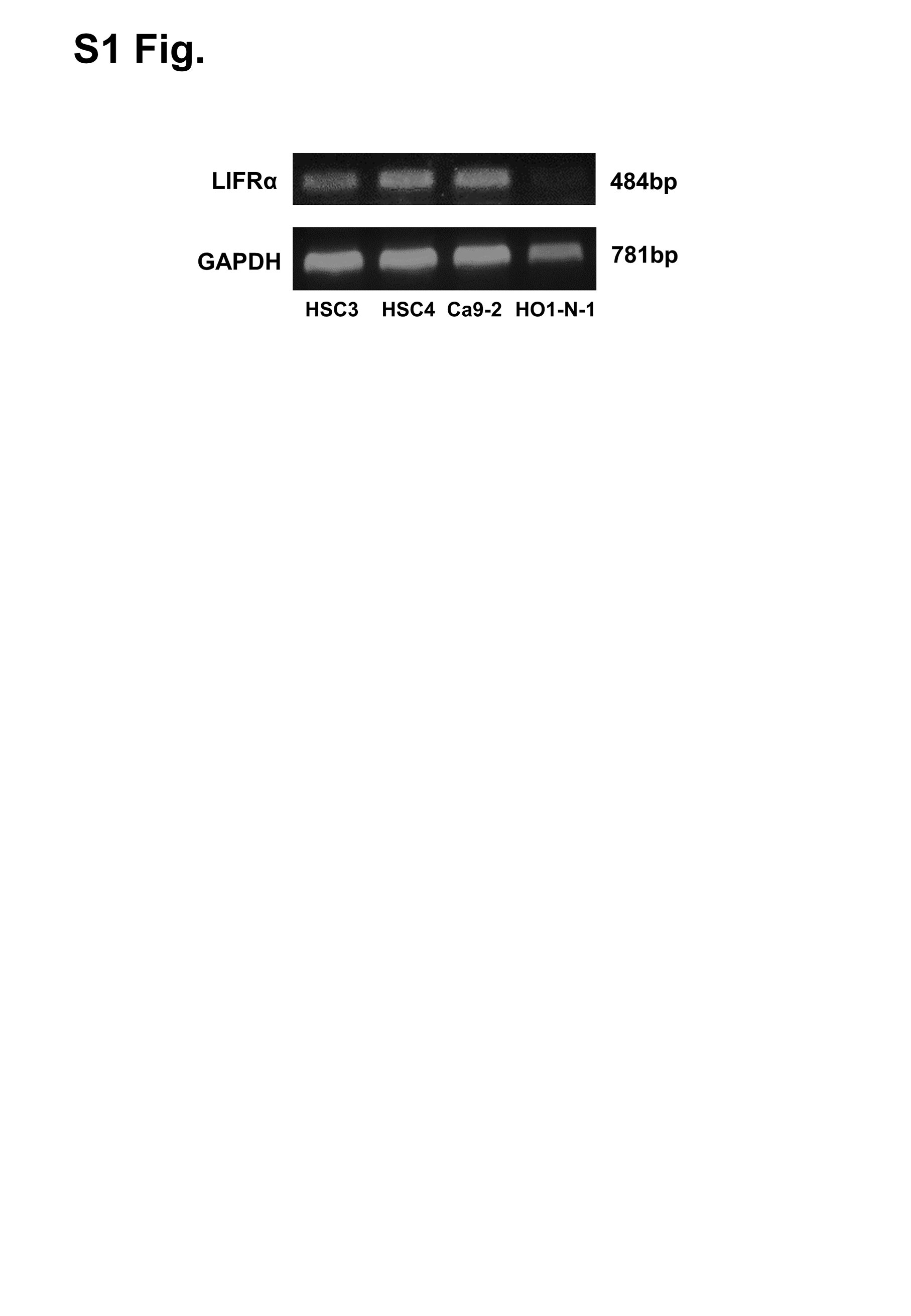

Supplement: S1 Fig — LIFRα expression in OSCC cells was detected using RT-PCR. The targeted sequences were amplified using Prime STAR GXL DNA Polymerase (Takara Bio, Shiga, Japan) and this amplification protocol: initial denaturation at 94°C for 2 min, followed by 35 cycles of 94°C for 30 s, 55°C for 1 min, and 72°C for 90 s, and a final extension at 72°C for 2 min. Primer sequences are shown in S1 Table. (TIF) [file pone.0191865.s001.tif]
